# Abstract of a Lecture upon Pathology

**Published:** 1873-02

**Authors:** H. R. Noel

**Affiliations:** Professor of Physiology and Pathology in the Baltimore College of Dental Surgery.


AMERICAN JOURNAL

OF

DENTAL SCIENCE.

Vol. VI. THIRD SERIES--FEBRUARY, 1873. No. 10.

### ARTICLE I.

*Abstract of a Lecture upon Pathology.*—[Continued.]

BY H. R. NOEL, M. D.

Professor of Physiology and Pathology in the Baltimore College of Dental Surgery.

This corpuscle, or mass of germinal matter, proto-plasm, or whatever you may decide to call it, has not only the power, when abnormally acted upon, of producing pus, mucus, and fibrin, but it has also other and most extraordinary manifestations of life. Now it is to be understood that the germinal matter of tissues and the "white blood corpuscles," are in our opinion identical, and whatever can be predicated of the one is equally true of the other, with this reservation, viz: Rapid propagation of self, though the rule in tissue troubles is the exception in blood troubles; and the tissue corpuscle may rapidly propagate, but the blood corpuscle rarely if ever propagates. Therefore, we do not believe in what is called Pyœmia, or pus in the blood, unless there should be an opening of the veins, as may occur after surgical operations, and thus direct absorption take place; in such cases pus may possibly be taken up by the blood vessels and carried into the general circulation, and carried in sufficient amount to induce the condition known as Pyœmia, which is attended by great and most dangerous prostration of vitality of the system.

But to return to the strange and most extraordinary manifestations of life which the germinal matter, more especially as the white blood corpuscle, is capable of giving. And we owe to Augustus Waller, of England—Cohenheim, of Berlin—Stricker, of Vienna—and some few recent observers, our present knowledge of the action of this corpuscle, or mass of germinal matter.

The observations of Waller were published in 1846, and more recently those of Cohenheim, Stricker and others. These experiments and observations prove that this corpuscle has the power of distinct movements; that it can elongate a portion of its body into the semblance of a foot, and retract it again; that it may move its position by its own power, and perform such movements as are known to be characteristic of a microscopically minute animalcule, called the Amœba. This animalcule is called by comparative anatomists a Rhizopod, or root-footed animal, and they give several sub-divisions of them, all characterized by the power of extending a portion of the body into a foot or root like prolongation, and then retracting this root or foot like extension of the body.

These movements of the Amœba, closely examined under the microscope, and the parallel movements of the "germinal matter," have led physiologists to class the movements of germinal matter as "Amœboid movements," and all accurate observers give to germinal matter the same powers granted Amœba, so far as movement is involved.

Now as regards the mass of germinal matter known as the "white blood corpuscle," the observations of the authorities cited are decidedly in favor of its Amœboid movements —and not only in favor of these movements, but almost conclusive as to the "migrations of the corpuscles." In other words, they affirm that the corpuscle does leave the capillaries and veins, and roams around in the tissues. The conditions requisite to this exhibition of its powers are simply obstruction of calibre of blood vessels as by ligature, or the production of inflammation by artificial means.

The experiments of Waller in 1846 were conducted upon the tongue and mesentery of the frog, and established two facts: 1st. The passage through the vessels of the corpuscles. 2d. The immediate closure of the wall of the vessel after the passage of the corpuscle, and this was true of *red blood corpuscles* also.

Subsequent experiments by Stricker and by Prussak proved that this migration of corpuscles could be induced by other causes than ligation of blood vessels, and could be produced in a circulation mechanically unobstructed; they proved that certain pathological conditions would induce the migration; or possibly these migrations induced many pathological conditions. They injected a solution of common table salt under the skin of a frog, and found that instantly an artificial scurvy was induced, and there were hemorrhagic effusions at various points in the frog, but that these effusions were simply "migrations of the red corpuscles," which had passed through the walls of the capillaries and veins. Waller, Cohnhein, Stricker, &c., all describe this passage of corpuscles through the walls, and in their experiments showed the corpuscles entering, half through and most certainly through the walls; the experiments giving examples of each and every position, from mere close proximity of corpuscle to wall, to the absolute passage through and safe arrival of corpuscle in the tissues extra vascular. Cohnheim thinks that there are "stomata," or minute openings in the capillary walls, through which these corpuscles pass, but Stricker and others have demonstrated that such is not the case, but that they pass directly through the walls, which, like an elastic structure, closes immediately behind them.

Waller proved the identity of "white blood" corpuscles, mucus corpuscles and pus corpuscles, and this was done in 1846—yet at this present time the very professors and teachers in our professional schools are unwilling to acknowledge its truth. Waller also proved in 1846 that the migrations of the corpuscles were not dependent upon the life of the animal experimented upon, for they occurred sometimes after the death of the animal, and the movements of the corpuscles were in every respect independent and apparently life-like, even after the animal's death—showing that the corpuscles possessed a life distinct from the animal, and that their life lasted sometime after the death of the animal. Now this "corpuscle called germinal matter," "white blood corpuscle," &c., which possesses the power of amœbiform movements, and which has life for a definite period after the body dies, and even executes movements after the death of the animal is certain, what is it? Is it an animalcule of the sub-kingdom Protozoa, and of the class Phizopoda? Has it any relation to the Porifera of the sponge family, or to the Zoophyta, with its division of Coralidœ? &c. In other words, shall we consider it as an animalcule, and place it with those animalcules which build sponges, and which make coral—even coral reefs and islands? And if this be an animalcule, does it build the osseous tissue, muscular tissue, nerve tissue, and every tissue of the human body as the Porifera construct the fabric of the sponge, and the Zoophyta construct coral reefs and islands? Remember we are to acknowledge this corpuscle as the active working agent in all histogenitic processes, vegetable and animal, and the tissue building of the vegetable and of the animal, so far as mere constructure is concerned, are one and the same. The process being the same in the two kingdoms, vegetable and animal, if, therefore, the agent—i. e. "corpuscle," be an animalcule in the one, it is an animalcule in the other, and the building of a tree, the building of a sponge, the building of a coral reef, the building of a human being, are so very nearly the same that we at present shall ignore any difference.

Now we are well aware that such ideas are at present *rank heresies* in the medical profession, but nevertheless we shall teach them, and shall tenaciously adhere to them until further light is given us, and when true investigation shall have demonstrated the error of such views, we shall most certainly abandon them, but not before this has been most thoroughly accomplished.

In Waller's experiments in 1846, he decides positively in favor of the identity of the white blood corpuscle, and mucus corpuscle, &c. In some of the experiments of Cohenheim and Stricker, the effort has been made to demonstrate the origin of mucus and pus corpuscles from the white blood corpuscles by migrations of these corpuscles through the capillary walls, and their aggregations in localized groups, or circumscribed communities, if you choose. If, however, we accept with Beale, the identity of white blood corpuscles, mucus corpuscles, pus corpuscles, and the germinal matter of tissue or tissue corpuscles, then pus formations are not necessarily the migrations of these white corpuscles from the capillary blood-vessels, but pus formations are clearly possible from similar aggregations of tissue corpuscles, or masses of germinal matter, called nuclei by the old writers, and cells by Virchow.

If the identity of all of these corpuscles be proven, then whatever is predicable of the one may be also predicable of each variety of corpuscle; and if white blood corpuscles be migratory, then are connective tissue corpuscles also migratory; mucus corpuscles migratory, and all masses of germinal matter in tissues migratory ; i. e. they possess the power of amœbiform movements. Now the question again comes up: Are all of these varieties of corpuscles "Animalcules?" and if so, should they not be classified? There mere anatomical outline is no sort of index as to their physiological or pathological powers; the bone corpuscles, which construct osseous tissue, and the *bone cancer corpuscles*, which construct bone and cancer together, are not by microscopic observation a this day demonstrably different, and yet how terribly different their physiological and pathological powers. If these corpuscles be animalcules, and if they possess these migratory tendencies, it would be well for us to examine most carefully, patiently and thoroughly those varieties which are found most frequently in what are called "Pus Formations," "Mucus Formations," "Fibrin Formations," &c. We might possibly, by patient inquiry, be ultimately able to show at once the status of the corpuscle or animalcule examined, and if a dangerous one, why we would at sight diagnose a wandering "bone corpusele or animalcule," or an "epithelial corpuscle or animalcule," or a much dreaded "cancer animalcule," or worse still—the first arrival of the cholera corpuscles or animalcules. Now we do not say that this happy solution of our trouble is near, and it may be years and years before we do solve these questions, but the tendency of modern investigation towards specialization in such studies is our hope for the future; the day may yet come when some acute observer shall bring order out of this chaotic mass of facts, theories, &c.; and as Harvey taught us the circulation of the blood, teach us the theory or true history of corpuscular migrations and defferential diagnosis between physiological and pathological corpuscles or animalcules.

We have stated that the "red blood corpuscle" is also migratory, and if a vein be ligated, and its return to the heart be interrupted through the usual channel or highway, it will also perforate the walls of the vessels and become a wandering stranger among the connective tissue corpuscles, and probably explore the country for an open route back to the heart and lungs. Be this as it may, the red corpuscle most certainly becomes occasionaly a nomad, and we will class it among the other animalcules.

You will at a glance perceive that as soon as the theory of the animalcular character of corpuscles is distinctly proven, we shall have to modify our present theories of pus mucus and fibrin formations, and we confess to a perfect readiness to do so whenever the data are sufficient to justify the change of views; until this be done, however, we propose to retain the views advanced in the first portion of our lecture.

For should we at this time, with our present information upon the subject, adopt the "animalcular migration" theory of certain diseases, then a congestion would be simply "a public meeting of the red blood corpuscles," or animalcules; and an inflammation would be an insurrectionary mob of the said corpuscles; while a clearly marked case of confluent small-pox would be considered as a general stampede of the white corpuscles from the centre to periphery and their collection first in distinct bodies, and afterwards as reinforcements arrived, their gradual occupation of the entire cutaneous surface.

Now we do not think this view tenable, but in a future lecture upon inflammation, we shall give you a modification of this theory of migrations, and deduce certain conclusions from facts which we consider substantiated.

---

